# Stepping into the virtual ward: a qualitative study on first-year residents’ experiences with VR simulation

**DOI:** 10.1186/s12909-026-08729-3

**Published:** 2026-02-07

**Authors:** Marte Mosling, Julie Horn, Athanasios Xanthoulis

**Affiliations:** 1 Department of Clinical and Molecular Medicine, Norwegian University of Science and Technology, NTNU, Trondheim, Norway; 2 Department of Surgery, Levanger Hospital, Nord-Trøndelag Hospital Trust, Kirkegata 2, 7601 Levanger, Norway; 3 Department of Obstetrics and Gynecology, Levanger Hospital, Nord- Trøndelag Hospital Trust, Levanger, Norway; 4 Department of Public Health and Nursing, Norwegian University of Science and Technology, NTNU, Trondheim, Norway

**Keywords:** Medical education, Postgraduate training, Qualitative research, Simulation, Virtual reality

## Abstract

**Background:**

VR simulation has been suggested as a resource-efficient tool in the education and training of doctors. Few studies have been conducted on what motivates different groups to use VR simulation, particularly during the transition from undergraduate to postgraduate learning. A deeper understanding of first-year residents’ experiences and attitudes may improve the development of VR simulation as an effective learning tool during this transitional phase. Therefore, the aim of this study was to explore the attitudes of Norwegian first-year residents towards this well-established yet rapidly evolving technology and their experiences of using it.

**Methods:**

First-year residents at Levanger Hospital, Norway were offered VR-based simulation from October 2022 to August 2023. Data were collected using semi-structured focus group discussions and in-depth individual interviews. Eleven first-year residents were interviewed. Data were analyzed using reflexive thematic analysis. Our results were interpreted in the light of Self-determination theory to better understand the influence of motivational factors on actual usage of VR simulation.

**Results:**

Three themes were generated from the data: (1) The playground of emergency medicine: Learning in a safe environment, (2) Barriers to be overcome and (3) Recognizing one’s reality in a virtual world. The findings showed that the participants considered VR simulation to be a safe and flexible learning tool for training in emergency care. The data also revealed various physical and psychological factors as barriers to using the tool. Variation, adaptation to individual needs and realistic experiences in VR simulations appeared to be important factors relevant to the clinical work of first-year residents.

**Conclusions:**

Our findings suggest that facilitating the use of VR simulation and offering content relevant to the target group may increase motivation. In this study, the perceived transferability from VR simulation to clinical practice appeared to influence its successful implementation and use. Future research should further explore applicability, the effect of incorporating learning theory into VR simulation software development, and the role of VR simulation in soft skills training.

**Supplementary Information:**

The online version contains supplementary material available at 10.1186/s12909-026-08729-3.

## Background

 Newly qualified doctors report feeling unprepared for several aspects of clinical practice and call for closer supervision and guidance, especially in emergency situations [1, 2]. First-year residents are still in training and need effective learning methods to become good clinicians. The overarching goal of medical education is to develop competent healthcare personnel who care about their patients and provide safe treatment. How to achieve this goal is seen as work in progress, involving several challenges [3]. A greater demand for healthcare workers, as well as increasing requirements for training hours, limited patient encounters, and a heightened focus on patient safety imply exploring new opportunities and developing existing technology to provide a standard curriculum [3].

In recent years, virtual reality (VR) technology has been increasingly used in various fields, including healthcare, the military, and education [4]. Interest in VR technology in higher education has been growing and it is thought to be full of promise, but many also question how well developed it is at this point in time [5]. It is argued that a combination of VR-based learning and face-to-face clinical practice may represent the future education of healthcare workers [2]. It is considered to be a useful complement to physical simulation, which is often resource-intensive, inflexible and requires a greater degree of organization. It can also enhance patient safety by providing training without creating potentially dangerous situations for patients [6, 7]. Further, VR simulation has been proposed as a more cost-effective learning method than physical simulation [8]. The software currently available is more advanced, cheaper and engages users at a level previously only possible in expensive simulators [6].

In healthcare today, there is an increasing demand for personnel in addition to higher qualification requirements. In this context, VR simulation has the potential to be a resource-efficient and practical learning tool. Understanding how it can be effectively implemented and refined to support the translation of theoretical knowledge into clinical competence requires qualitative explorations of learners´ attitudes and lived experiences. Multiple studies have explored the use of VR in undergraduate medical education and professional development within respective fields of specialization [6, 9–11]. However, only a limited number of studies have examined the use of VR simulation among first-year residents during the transitional phase from undergraduate to postgraduate training – a period that is particularly critical for shaping clinical identity, building confidence and adapting to new responsibilities [12]. The aim of this study was to investigate first-year residents´ experiences of and attitudes towards the use of VR-based simulation as a learning method in Norway.

## Methods

The first-year residency is a one-year mandatory postgraduate hospital training that all newly qualified doctors in Norway must complete before choosing a particular field of specialization. The introduction of VR simulation at Levanger Hospital aimed to enhance learning for this group by simulating emergency medical cases. The VR simulation project would enable the first-year residents to safely improve their skills and experience in order to become better equipped to face clinical reality. VR simulation at Levanger Hospital was offered from October 2022 to August 2023. The simulation was delivered using head-mounted VR headsets (Meta Quest 2, MQ2), providing a fully immersive first-person experience. The VR software platform was delivered by Oxford Medical Simulation (OMS) and included 20 emergency medical cases from various disciplines. Participants interacted with the virtual environment in the scenarios using head movement and hand/controller input. Information about the offer and training in the use of the headsets and controllers was provided to all first-year residents at the beginning of their residency period. They were permitted to use the headsets freely within the hospital area and could collect them as needed from an office during working hours or in their free time.

### Design

The aim of this study was to investigate the experiences of and attitudes towards the use of VR-based simulation as a learning method for first-year residents. To gain deeper insight into this topic, we used semi-structured focus group discussions and in-depth individual interviews, depending on the participants’ preferences. Focus group discussions allow group dynamics to influence the topic under discussion, thus providing different information from individual interviews, as participants react to one another’s comments [13]. On the other hand, individual interviews offer more flexibility and no group pressure. By combining the two interview methods, we aimed to elicit more nuances, capture complementary perspectives, and to reach a sufficient number of participants. While larger groups would have been preferable, coordinating participants’ clinical schedules proved challenging, resulting in focus groups consisting of 2–3 participants.

### Participants and recruitment

All first-year residents at Levanger Hospital from January to August 2023 were invited to participate in the study. The total number of potential participants was 27, 13 of whom started their first-year residency in September 2022, while 14 started in March 2023. For the latter half of the first-year residents, the VR simulation was also integrated into various learning objectives, in the form of learning activities in the “competence portal”, which is a documentation system for the training of residents used throughout Norway [14]. All first-year residents had equal access to the VR simulation during the study period. They were contacted by email at the beginning of June 2023 with an information letter and an invitation to participate in the study attached. A reminder was sent out after two weeks to increase recruitment. Eleven agreed to participate, and seven interviews were conducted, five of which were individual in-depth interviews, while two were focus group discussions with two and three participants. There was one dropout due to the participant leaving the hospital and starting work in primary care.

### Data collection

The interview guide was developed by the research team and included questions on previous experience with VR simulation, thoughts about the offer of using it, its advantages and disadvantages and its perceived learning effect (see Appendix 1). The interviews were conducted by MM from June to November 2023. JH was present during the first interview and provided feedback. The interviews lasted from 15 to 40 min and were audio recorded. The researchers continuously assessed the number of participants needed and new participants were recruited until the information power was felt to be sufficient to address the research topic [15]. During the data collection phase, we continuously assessed the dataset’s ability to capture both the depth and variation of participants’ experiences [16, 17]. After conducting seven interviews, no new insights emerged, and we concluded that the dataset had achieved strong informational power, representing a broad spectrum of experiences.

Prior to the interview, the participants were given a short questionnaire involving demographic variables and previous experience of using VR (see Appendix 2). Demographic characteristics (age, sex and previous experience with VR) are presented to provide contextual information about the sample and illustrate variation within the participant group (Table 1). At the time of the interview, most participants had used the offer of VR for 1–4 h. We also included one participant who had not used the VR simulation at the time of the interview, because this interview also contributed important findings to the study.

**Table 1:** Participant characteristics (n = 10)

Characteristics	*n* (%)
Age	
< 30 years	6 (60%)
* ≥* 30 years	4 (40%)
Gender	
Female	7 (70%)
Male	3 (30%)
Started first-year residency program	
September 2022	4 (40%)
March 2023	6 (60%)
Previous use of VR headset in learning or work context	
Yes	2 (20%)
No	8 (80%)
Previous use of VR headset in other contexts, e.g., gaming	
Yes	3 (30%)
No	7 (70%)
Number of hours spent on VR simulation during the first-year residency VR simulation project	
0 h	1 (10%)
1–4 h	8 (80%)
5–10 h	1 (10%)

### Data analysis

We used an inductive approach involving Braun and Clarke’s six steps for analysis and interpretation of data. Reflexive thematic analysis enables systematic data analysis, while also providing sufficient flexibility in the analytic process. It is a robust and easily accessible method that is well suited to researchers unfamiliar with qualitative analysis [17]. As the first step in the analytic process, the recordings were transcribed by MM, who also performed the analysis. The recordings were listened to several times to ensure correct transcription. The interviews were anonymized and the participants were given pseudonyms. The second step involved a new examination of the data, where excerpts were marked with codes. The third step of the analysis was to find common features and patterns in the different codes, and then group these under different initial themes. This process was dynamic, and initial codes and themes were altered several times. A reflection book and a code book were used to ensure that no important information or reflections were lost during the process. The research team members worked individually and in planned collaboration meetings. Step four of the analysis involved examining whether the data were still meaningful in relation to the content of the themes, followed by the beginning of sorting according to themes. Here, the focus was on ensuring that the meaning of the various statements in the interviews had not changed during the analysis [17]. This was done by examining the quotes in the context in which they were spoken. Steps five and six consisted of analyzing each theme separately, writing definitions of the themes, and naming them. This process was time- consuming, as it was important to create clear definitions. The analytical narrative was then refined and illustrative excerpts from the interviews were selected. During analysis, we identified several motivational factors influencing the use of VR simulation that aligned with central concepts of Self-Determination Theory (SDT) [18, 19]. SDT was not used to guide data collection, coding, or theme development, but was applied post hoc to support interpretation of these factors.

### Self-determination theory

Self-determination theory, developed by Edward Deci and Richard Ryan, is among the major motivational theories in psychology [20]. Previous studies suggest that this might provide a useful theoretical framework when examining user motivation in a virtual reality context [18, 19, 21]. One of its seven principles is the distinction of three innate needs that fuel psychological growth: (1) Competence, (2) Autonomy and (3) Relatedness [22]. In brief, the need for competence refers to the desire to feel effective in the actions you perform, while the need for autonomy refers to the desire to be your own source for behavior. The need for relatedness refers to the desire to feel connected to and care for others, as well as being cared for. It can also mean being accepted or belonging to a community [22]. The first-year residents need to take responsibility for their own learning and autonomously acquire a habit of spending time and effort to keep up with medical knowledge and expand their clinical competence [12]. SDT seems particularly relevant for understanding what motivates newly qualified doctors in adapting to their new roles when engaging with new learning tools such as VR simulation.

### The research team

The research team consisted of a female fifth-year medical student at the Norwegian University of Science and Technology (MM) with no previous research experience, and two supervisors employed by Levanger Hospital: a male gastrointestinal surgeon (AX) and a female gynecologist and obstetrician (JH). The two supervisors teach medical students in their respective fields and each holds a doctoral degree. AX initiated the introduction of VR simulation at Levanger Hospital, which was one of the reasons why the team wanted to examine its effect and how users responded to it. AX did not participate in the recruitment of participants or the data collection and had no formal administrative or supervisory role in the education program of first-year residents. JH has extensive experience in qualitative research. MM was in clinical practice at the time of the last interviews and had recent experience of interdisciplinary VR simulation with nursing students. They used the same equipment and had access to the same cases as the participants in this study. This provided greater insight into potentially different experiences of the participants during the simulation and improved understanding of the challenges involved. The research group brought different personal and educational experiences with VR to this project, potentially enriching the reflexivity of the analytic process.

## Results

The main findings of the study were that the participants described VR simulation as a safe and flexible learning tool to practice dealing with emergency situations, but several external and internal barriers prevented them from using the equipment more regularly. Realistic simulation was highlighted as an important motivating factor for prioritizing VR simulation. We generated the following three themes to describe the participants’ experiences of using VR simulation: (1) The playground of emergency medicine: Learning in a safe environment, (2) Barriers to be overcome, and (3) Recognizing one’s reality in a virtual world.

### Theme 1: the playground of emergency medicine: learning in a safe environment

The theme *The playground of emergency medicine: Learning in a safe environment* reflects a gamified approach to VR simulation. Most of the participants had little previous experience with VR headsets, and it was mainly related to games and entertainment. Some of them were skeptical of the use of VR simulation as a learning tool before using it themselves, mainly because they did not think it would be realistic.

Participants who had previously used VR headsets were less surprised by the design of the simulation than those with little experience. This affected their expectations prior to using the simulation. The participants with less experience were surprised at how realistic they found it, and also reported finding it entertaining and engaging. Those with more experience felt that the simulation was consistent with their expectations. The participants expressed disappointment about the limited interactivity and about the fact that the possibilities offered by VR simulation were not utilized to a greater extent. Some of the participants stated that they had low expectations of learning outcomes and regarded VR simulation to be closer to entertainment. However, the majority had a positive attitude and expected it to make an important contribution to learning in the future. One of the participants who had the most knowledge and interest in gaming said the following:

“I think it [VR simulation] is the future. It’s very simple and very easily accessible, but it has potential for improvement. VR is going to be part of the future. It’s getting better and better, I can already see how much they’ve developed it in games and how realistic it’s become. So, if you get the same level of quality in these simulations, it’ll be really cool.” (Anne).

There seemed to be some variation in the extent to which the participants appreciated simulation as an educational tool in general due to individual factors influencing favored learning methods. Some felt a need for control, structure, and a slow pace with time to think in order to benefit from learning. Others liked the hectic pace and the opportunity to test their reactions and what they remembered in a stressful situation. The participants found it difficult to determine how directly the VR simulation was linked to learning outcomes. It was a positive learning experience to practice clinical thinking by making their own decisions, and this was also felt to be part of the everyday work of more experienced doctors. The participants reported feeling safe in the simulation and dared to challenge themselves more than they would have done in a real emergency situation. They appreciated practicing short, targeted emergency medical cases of their own choosing, in order to establish good routines in preparation for their future emergency care. This enhanced their motivation by providing confidence and they found it a useful way of learning through repetition with the aim of becoming a safer and more effective clinician. Some participants said that the excitement of the cases wore off as they gained experience and that the feeling of learning something new to increase their competence was an important factor in how rewarding they found VR simulations. In other words, they were less motivated by the enjoyment aspect of learning through VR simulation than by the perceived learning outcome.

The participants said that they often chose cases at random, thus not necessarily dependent on whether they were on a surgical, medical, or psychiatric rotation. They felt that they needed a great deal of practice, which meant that it did not matter which simulation scenario they chose. Others were motivated through a sense of relatedness and decided on the basis of the rotation they were on to help them prepare for what they might encounter later, or to practice useful skills they needed. Several participants avoided topics they felt unsure about. They acknowledged that they needed more practice in those areas, but found it difficult to get started because they felt unable to master the skills involved. As recent graduates, they had not had time to build up an extensive toolbox of clinical skills and they found it useful to acquire more tools for everyday use through VR simulation. They explained that they learned more from making mistakes and found it easier to remember the occasions when they had done badly in simulation. The participants’ goal was to avoid making mistakes with real patients and their focus was clearly on ensuring patient safety. They wanted to become better clinicians, to the benefit of patients.

The participants described situations where they felt uncertain, often about what they considered to be simple issues, and wished they could have practiced these before starting a duty period. One example was intravenous fluid administration, which they were often asked about when they went to the emergency department. They found that the hectic atmosphere there left little time to practice what they felt unsure about. Emergency care was what the participants found most stressful in their everyday work as first-year residents, but they believed that VR simulation could be an effective method to boost their confidence. Most of them regarded VR simulation as useful preparation for their work at the emergency department, or as part of their medical studies when access to real patients was more limited. They felt that they had less responsibility as medical students, but also more time and energy to use VR simulation. The participants stated that at the start of their practice period in their fifth year of medical school, they were daunted by the idea of taking on the role of a doctor for the first time, and therefore suggested this as a suitable time to introduce VR simulation. They felt that it would be a useful way to increase their confidence in an unfamiliar and challenging setting. One of the participants explained:

“[…] it [VR simulation] must be very useful for medical students, not just for first-year residents. Playing a bit, making things seem less scary. […] I think that’s the problem for a lot of people starting their first-year residency. […] They’re often afraid, like: ‘Oh my God, there’ll be so much stress and scary cases’, but if you’ve had the chance to play around with it beforehand, with VR for example, which you can do much more easily than simulating with a whole bunch of people. […] It might make things a bit less frightening and make people feel more relaxed before they go to the emergency ward.” (Sara).

### Theme 2: barriers to be overcome

The theme *Barriers to be overcome* explores external and internal factors that influenced participants’ decisions on whether or not to use VR simulation. In the interviews, they indicated that they would generally like to use it more frequently than they had been able to. External factors such as equipment, space and availability came in addition to other tasks that competed for their time. This increased the barrier to using VR simulation. Physical discomfort including headache, nausea and dizziness -otherwise known as cybersickness- due to the use of a VR headset was a major barrier for some participants. They had to plan their days differently and take into account reduced performance after use. They found the headset too tight and felt exhausted after being in the simulation. They mentioned low resolution as one of the reasons for this. For some, the biggest challenge was the lack of compatibility with their glasses. For those who used prescription glasses, this was a major factor in their decision not to use the headset. One of the participants said:

“When I was wearing the headset, I didn’t notice anything in particular, but as soon as I took it off, I had an intense headache. […] I had to take off my glasses, but then I could see worse in the simulation. That may be part of why I felt nauseous, because I was squinting at things […].” (Hans).

Several technical and logistical issues hampered the use of VR simulation among first-year residents. Problems in physically adjusting the headset, wasting time on configuring software settings and logging in were mentioned as key barriers. This was especially true for those who considered themselves to have poor technical skills, but it was seen that repeated use helped to mitigate this barrier.

Another logistical factor that played a part was access to a suitable room. The participants said that a feeling of discomfort when using the VR simulation in an open office environment could represent a further barrier to using it.

Noise could affect both the person using the simulation and people doing other work in the same room. One participant said:

“I really think you could have a room where you could have it [the VR headset] […] in the medical room [workroom for first-year residents in the medical department] it’s quite cramped with lots of chairs and at the end of the day there are a lot of people sitting there writing, so it can get a bit noisy. […] It’s hard to hear what’s going on. I think accessibility is important. […] It should be emphasized from time to time, I think people forget about it and make it a low priority.” (Beate).

A further barrier was an inadequate system for borrowing the equipment, such as a lack of procedures for recharging, borrowing on an hourly instead of a daily basis, and a ban on using it outside the hospital. The participants pointed out the advantage of having headsets easily accessible, as this would reduce the time spent.

Although the VR simulation training project was intended for first-year residents regardless of their rotation stage, its usage was shaped by organizational culture. First-year residents rotate between different departments; they noticed a considerable difference between them and felt that it could increase the use of the simulation if all departments had a sense of ownership of it. One of the participants said:

“[…] I think it would help those of us in internal medicine if there were [headsets] a bit closer to us. It sounds silly, we just have to go down one floor to an office. […] I didn’t quite know where his office [the senior physician who kept the headsets] was at first. […] I don’t know any of the senior doctors there. They don’t know me. […] It was a bit more of a barrier for us in internal medicine.” (Helene).

The participants found that the surgical department had greater ownership of the headsets, as the initiative mainly came from there. They stated that there was less of a barrier when they rotated there and became more familiar with the staff in that department. They wished they had started using the VR simulation earlier, as they felt that they would have benefited more from regular use. Those who had attended the introductory week for first-year residents where they were all introduced to the headsets felt that it was an important factor in reducing the barrier to using VR simulation. They were given access to a Microsoft Teams page and considered this to be a useful information channel, in addition to receiving reminders by email. They also tried to remind each other to use the headset and felt there was a great need for reminders, as this was a relatively new learning option. A lack of visibility meant that it was soon forgotten. Having a person they respected and looked up to promoting the use of VR headset could encourage greater use and lead to higher expectations for learning outcomes. They mentioned the lack of promotion of the headset in the medical department as a barrier to using it. Some of the participants said that they tried to encourage others in using VR simulation, because they felt it was important and they were afraid it would be discontinued. Those who used it most frequently were positive about the benefits and wanted more people to make use of it.

The first-year residents had busy days, and VR simulation competed with work on the wards, in the emergency room and in the emergency surgical clinic. Their workload was difficult to predict, some days being very busy and others relatively quiet. However, the total amount of work meant that there was always something they could do to help, making it difficult to justify using VR simulation instead of relieving their colleagues. Simulation had low priority when they felt it was less useful than working with real patients. Given the choice between treating real patients or using VR simulation, they chose the former. Unpredictable working days made it difficult for some to prioritize VR simulation. One participant put it this way:

“Some days are very busy, you feel like you’re working 150%. If there’s a quiet day, many of us want to relax a bit, too. So, I can understand if people don’t want to prioritize VR.” (Sara).

The participants thus found it difficult to find time for VR simulation and described it as a luxury project. However, most of them were pleased that it was available and wanted to recommend it to the next group of first-year residents. Some suggested setting aside time in the form of individual training days when VR simulation could be used, while others said they still would not use it even if they had more time. Their argument was low transferability and little benefit, based on the design of the simulation at the time. The lack of time set aside for VR simulation was a further barrier to using it. One of the participants said the following:

“When it [the VR simulation] was first rolled out, I tended to think I didn’t have much faith in the learning benefits, because I didn’t think we’d have time to do it, and that was true to some extent.” (Kasper).

The amount of learning and the personal feeling of benefit from human interaction were decisive for whether the first-year residents chose to prioritize VR simulation. For some, what made their working days meaningful was interaction with real patients, which was lacking in the simulation. One participant said:

“I like meeting patients. I think writing is just so boring. I think it’s fun to talk to people and it gives me something, on a personal level. And you don’t get that with a VR meeting. So, if I had to choose…, but that has nothing to do with the learning benefit.” (Beate).

For the second group of first-year residents, the VR project was included in the learning objectives as motivation for increased use. The participants felt differently about this; some saw it as a form of pressure with reduced autonomy, while others found it a useful measure to justify greater use and an enjoyable way to learn. The participants said that the learning objectives were diffuse; they wanted to make them more specific. They therefore suggested a list of recommended cases linked to relevant learning objectives. They prioritized documenting that they had fulfilled the mandatory learning objectives before commencing the learning activities related to VR simulation. Although they preferred physical simulation, most said that VR simulation was a useful supplement and they appreciated the flexibility it provided, enhancing their sense of autonomy. They described VR simulation as saving space and being easily accessible, requiring little organization and staff. They also had the impression that it saved time and was cheaper over the long term. Everyone interviewed had used the headset during working hours and most of them said that they did not want to spend any of their free time on VR simulation. Some felt that more simulation options should have been offered, while others were satisfied with what was available.

### Theme 3: recognizing one’s reality in a virtual world

The theme *Recognizing one’s reality in a virtual world* concerns the importance of feeling drawn into the VR simulation for the level of engagement. Some felt that they were too passive and missed more interactivity in making diagnoses and performing practical tasks. Several mentioned background noise and the design of the emergency room in the simulation as factors that provided a more realistic experience. The participants stated that the VR software captured the workflow in an emergency situation and focused on assessment, treatment and the consequences of the choices made. It gave them a lift to realize that the choices they make have consequences and they felt that this made them more effective. They wanted to be efficient but still do a thorough job; this balance was particularly difficult to achieve for first-year residents with limited experience. One of the participants elaborated:

“I think you learn how to assess a patient quite quickly. […] But you mustn’t miss or overlook anything either. I thought that was quite good, because I think, at least in your first year, you tend to spend some time and be extra careful. The software actually teaches you quite well that there are important things you mustn’t miss, but you also have to keep an eye on the clock to be effective in what you do.” (Helen).

The participants reported having limited time in the simulation, which was a stress factor. They found that they could not always keep to their routines, which made them skip questions and thus lose points. Several said that the score they achieved made for a less realistic experience and reduced motivation, because they lost points for actions that were correct according to the Norwegian guidelines. However, basic elements in the simulation such as medical history, physical examination and targeted treatment were perceived as well-designed and useful. Some said that feeling stressed and under pressure was an important part of the learning process. They found it difficult to realize what they did not know until they were under pressure and had to react automatically. Some participants saw the value in being unprepared; they could then explore different approaches and thus learn more. They argued that they never knew what they might encounter in their everyday clinical work, which made the VR learning more realistic. One of them said:

“[…] the most important thing is that you give fluids when that’s indicated, and that you see the effect of your actions. And afterwards you go through what you did well and what you’ve forgotten. So that you can review it and memorize it for a real patient[…] so it’s the transferability that’s useful. As it helps me to be a better clinician.” (Kari).

The participants pointed out that throughout their studies and work they needed to be challenged in different areas and that this was constantly changing. The VR simulation had to match these changing needs to be seen as meaningful. The timing of the introduction to VR simulation was thus important for the perceived benefits. Some felt that the cases did not challenge them sufficiently and would prefer working with rarer diseases, while others found it useful to practice what they would probably encounter in their everyday work. Having scenarios at the right level of difficulty was a decisive factor in whether they were motivated to use the VR simulation or not. One of the interviewees described it as follows:

“It’s similar to when you start a job, then you spend a lot of time in the emergency room. So, then I thought you learn more from being in emergency than sitting there with your headset on. I think I would have expected more in terms of learning outcomes if I’d used it as a student.” (Emilie).

Several of the participants said that they missed receiving feedback on their work. Importantly, the feedback had to be relevant, and some of them stated that the VR simulation did not help them in the areas they wanted feedback on. Here, they mentioned patient communication and examination techniques. Some felt that the feedback they received in the VR simulation was unnatural in the given situation, such as losing points for not having washed their hands before talking to a patient. However, they also said that one of the things they appreciated about simulation in general was detailed feedback. One of them expressed this as follows:

“[…] you’re still supposed to be in an educational setting and getting feedback, but it doesn’t happen that often that senior doctors tell you: ‘That was a useful medical history’ or ‘You examined that patient well’[…] you don’t get much of that [feedback] […] but in simulation, the whole point is that people are objective and give you feedback.” (Helene).

Participants mentioned that the on-call attending consultant function made the VR simulation feel more realistic. They pointed out that the flowcharts and the lack of possibility of taking notes during briefing and handover pulled them out of the simulation. They said that the on-call attending was more present in real life, but that the possibility of consultation calls by phone provided greater independence. However, they still felt somewhat frustrated with the design of the function, as described by one participant:

“[…] you call the on-call attending inside your headset and then you get a huge list of ‘Remember to do this. Remember to do that […]. And you can’t note it all down while you’re inside the headset, so I’m sitting there trying to remember everything. And it’s pretty difficult. Well, you can always check the guidelines, so I open up the guidelines and then it’s like ‘How am I supposed to interpret this?’” (Hans).

The participants considered it important that the diagnosis and treatment were of good quality because this determined whether the simulation could be transferred to everyday clinical work. Most of them emphasized differences in Norwegian and British guidelines as an example of this. They would rather use an updated version of the software adapted to the Norwegian guidelines and practice in order to increase their sense of competence.

## Discussion

Knowles’ concept of andragogy applied to postgraduate medical education states that physicians in training are motivated by their personal goal of becoming skilled professionals [23]. This was explicitly stated also by our participants. Our findings show that the participants generally had a positive and forward-looking attitude to VR simulation but varied in their use of the tool. Some described low expectations for learning outcomes which influenced their initial motivation to try the VR simulation. Viewed in the light of SDT, our analysis suggests that the intrinsic motivation to use VR simulation depends on the extent to which it satisfies the residents’ needs for competence, autonomy, and relatedness. Similar studies have shown that VR simulation is used less than expected in education unless it is made mandatory, accessible and adapted to the skill level of its users [24, 25]. This also applied in our study and the reasons mentioned for limited use among our participants included discrepancies with Norwegian guidelines, insufficient interactivity, little variation in cases, physical discomfort and lack of time. This indicates that VR simulation should be better adapted to the target group and that users must find the learning outcomes relevant and rewarding to strengthen their intrinsic motivation.

A recent qualitative study on strategies for enhancing the quality of virtual education among medical students suggested a shift in learners’ expectations towards structured teaching practices, timely feedback and active student engagement [26]. Similarly, the first-year residents in our study requested increased interactivity, a more organized framework and opportunities for structured feedback. Previous studies have shown that implementation of VR simulation in education can be challenging with limited data on how to achieve it successfully [24, 27, 28]. Identification of barriers and facilitators, as well as strategies for addressing them, appears central to optimal utilization of resources [27]. This also involves investigating motivational factors for using VR simulation as explored in this study. The interviews revealed that a clear framework and time set aside could lower the threshold for using VR simulation, as was seen in a similar study involving newly graduated doctors [24]. Previous research on the features of simulation tools that lead to effective learning emphasizes the need for repetitive practice, increasing levels of difficulty and clinical variation [29]. These elements were pointed out by our participants who expressed a desire to become skilled clinicians, but were challenged at this stage by their limited experience. Experiential learning theory emphasizes learning through experience, reflection and improvement [30]. In this context, immersive VR simulation may deliver repeatable training experiences in a realistic and safe environment promoting contextual learning and competence development for first-year residents, a potential that has also been demonstrated in previous studies [31]. Our participants were motivated by the opportunity to practice cases as often as needed in order to become more efficient and competent clinicians. In our study, first-year residents’ individual strategies for coping with the transition from undergraduate to postgraduate training seemed to influence their case preferences in the VR simulation depending on how comfortable they felt with challenging themselves. Furthermore, the option to choose cases themselves appeared to affect their sense of autonomy and consequently increased their motivation. Enjoyment alone did not seem to sufficiently motivate their use of the VR simulation.

It has been observed that more active VR interventions appear to improve users’ knowledge and skills to a greater extent than less active alternatives [11, 32]. The engagement of the participants in the present study increased in proportion with the degree of realism in the simulation. VR simulation is viewed by medical students as closely comparable to advanced patient simulators, with the additional advantage of being engaging, immersive and portable [33]. The participants in our study stated that simulation software that is realistic and interactive can improve learning outcomes, which concurs with other studies in the field [2]. This also strengthened their intrinsic motivation by allowing them to focus on improving their skills. Applying gamification in the development of VR software is a versatile and increasingly theory-driven approach in educational settings [34]. A previous study involving surgical residents has shown that gamification of simulation software can encourage use by increasing external motivation. However, the researchers emphasized that limited use of VR simulation was not due to a lack of motivation or discipline, but rather to limited availability and competing tasks [35]. This is consistent with the findings of the present study where first-year residents experienced a hectic everyday workload with reduced autonomy, which in turn undermined their motivation for using the VR simulation. This highlights the importance of facilitating the use of VR simulation at the organizational level.

Including VR simulation in learning objectives seemed to decrease the motivation of some participants; this has been seen in other studies, where it was considered more as an item to be checked off than an engaging learning tool [24]. Our participants expressed similar feelings about it and motivation to use it may partly be dependent on the sense of autonomy involved. Giving first-year residents the opportunity to practice making decisions about their patients and supporting them as important members of a healthcare team can encourage autonomy in medical education and increase the sense of relatedness. This is a critical aspect of becoming an independent clinician [36]. Our participants missed trying things out for themselves in their everyday work life but appreciated this possibility in the VR simulation. Overall, these findings show how their perceived autonomy both decreased and increased through the VR simulation project in different ways.

Few studies have focused on the acquisition of soft skills such as communication or empathy in VR simulation [10]. The lack of authenticity and limitations in learning communication skills in VR simulation have been pointed out in previous studies [37]. Our participants similarly stated that they wanted to practice communication and called for more feedback on it in clinical situations. In this respect, they felt that they did not derive sufficient benefit from the VR simulation as it was designed at the time. A Norwegian study among resident doctors in child and adolescent psychiatry found that VR-based simulation was a suitable method for training soft-skills, and underscored the benefits of learning through observation and peer discussion [9]. Combining VR simulation with case discussions in small groups, ideally guided by an experienced clinical tutor, may enhance the educational value for first-year residents. The integration of artificial intelligence into VR simulation software may prove valuable for practicing soft skills in the future by creating adaptive and realistic scenarios responding dynamically to the learner’s actions [38]. This can allow for individualized training experiences, thus enhancing engagement and motivation.

Previous studies have suggested that educational frameworks or theories may be important when designing complex educational interventions. However, few studies have explored how VR-based medical simulation training can be strengthened based on specific learning theories [5]. The theoretical framework needs to integrate pedagogical theories and game design [39]. A scoping review on gamification in clinical reasoning education points to the underutilization of cognitive theories in gamification design [34]. The Cognitive load theory states that novices are overwhelmed by complexity and need sequenced learning [40, 41]. Scaffolding seems to be important in our study, as first-year residents can have different knowledge level and therefore need varied scenarios and degrees of difficulty. VR simulation might decrease the sense of being overwhelmed while also optimizing the learning load if the software is more individually tailored. Our study suggests that training clinical reasoning by providing methods for applying learned principles and developing cognitive schemas in VR simulation may be useful during the transitional phase of first-year residents. Further research is needed in investigating the effect of incorporating specific educational theories such as Cognitive load theory, Script theory and the Dual process theory to enhance the educational value of the simulation software [34].

To our knowledge, this is the first qualitative study focusing on VR simulation among first-year residents in a Scandinavian setting. The Scandinavian context is particularly relevant due to its distinct educational structure, emphasis on early clinical responsibility, and collaborative healthcare culture, which may influence how VR simulation is perceived and integrated into postgraduate training. While similar findings have been reported internationally, we believe these strengthen the credibility and the transferability of the findings in our study [24–26].

Norway is expanding its intake of medical students despite limited resources for postgraduate training [42]. Innovative tools like VR simulation hold significant potential to support and enhance clinical education.

### Implications and recommendations

This study, alongside other similar studies, may help guide hospitals considering implementing VR simulation training for their first-year residents as well as inform the development of VR simulation software. We suggest aligning the content of the VR software with local guidelines, providing structured feedback and gradually reducing guidance in pace with increasing skills. Ensuring adequate time, dedicated room and easy access to headsets appears to influence successful implementation.

### Study strengths and limitations

The sample showed good variation in terms of gender, age, and use of the VR simulation. To minimize the risk of self-selection bias, we clearly stated in the invitation that we aimed to recruit participants regardless of prior experience with VR. A medical student interviewing first-year residents may have influenced the interviewer-participant dynamics. This likely had a positive effect and fostered a deeper understanding by reducing power asymmetry and enabling the interviewer to interpret contextual information. Data were only collected from one hospital and are not necessarily representative of experiences in other Norwegian hospitals, which may have affected the generalizability of the findings. Only one professional group was interviewed and it might be interesting to study and compare other groups. The participants suggested medical students as a relevant group for the intervention. Future research could examine the impact of the recommended changes identified in the study. There seemed to be a correlation between the amount of use and the perceived benefit, which may mean that those who had made little use of VR simulation reported less benefit than those who had used it more. Some participants had only tried the simulation once during the introductory week and therefore probably did not have a clear idea of its potential. Despite this, those participants shared valuable experiences and reflections on why they decided against using the VR simulation. Although Self-determination theory was introduced post hoc during the analytic phase and was not used in development of the interview guide or the initial coding process, it proved useful in interpreting the motivational factors that emerged inductively from the data.

## Conclusions

The first-year residents described VR simulation as a helpful learning tool, yet they made limited use of it during the project.They suggested that future implementation of VR simulation should prioritize accessibility, a wider range of cases and dedicated time for training, preferably in a more structured way. We suggest that future studies explore further the role of VR simulation for soft skills training and its possible integration with in-person simulation to see if this may enhance learning outcomes and perceived relevance. Furthermore, we suggest investigating the incorporation of learning theory in the development of software to determine its effect on learning outcomes and motivation. It may be of interest for educational institutions and hospitals to contribute their experiences and to support Norwegian developers of medical VR simulation software. This might help solve the challenges mentioned and increase the benefit for the target groups.

## Supplementary Information

Supplementary Material 1.

## Data Availability

The datasets analyzed during the current study are not publicly available because the participants have not consented to sharing the interview material.

## Abbreviations

MQ2: Meta Quest 2
OMS: Oxford Medical Simulation
VR: Virtual reality
SDT: Self-Determination Theory
