# Supplementary material for: Stepping into the virtual ward: a qualitative study on first-year residents’ experiences with VR simulation
Source: BMC Med Educ. 2026 Feb 7;26:400. doi: 10.1186/s12909-026-08729-3 (PMC12977727; doi:10.1186/s12909-026-08729-3)
Supplement: Supplementary file 1 — Supplementary Material 1. [file 12909_2026_8729_MOESM1_ESM.docx]

# Appendix 1

Interview guide

**Information for participants at the beginning of the interview:**

- Thanks to the participants for taking the time to participate.
- Brief introduction of the interviewer, the background of the study, and its purpose.
- Inform the participants about the confidentiality applying for both the interviewer and assistant moderator.
- Ask participants to avoid mentioning names or identifiable background information, such as age, location, time, diagnoses, or specific events.
- All statements will be anonymized after data processing.
- There are no right or wrong answers. All experiences can be valuable.
- We are interested in experiences. Examples are welcome. The interviewer will help guide the discussion if it drifts too far from the research topic.
- The focus group discussion will last 60–90 minutes.

1. VR headsets are commonly used in a variety of contexts. What prior experience do you have with VR headsets?

1. Can you tell us a bit about your previous experiences with simulation-based training?
2. Including training with VR headsets?

1. What expectations did you have regarding VR simulation before you started?

1. Can you tell us a bit about why you may not have used the VR simulation?
2. Do you have any thoughts on what could have been done differently?

1. What experiences did you have using VR simulation during your first-year residency program?
2. How did you use the equipment? (alone, with others, during your working hours, in your free time)
3. What thoughts do you have on the learning outcome?
4. Advantages/challenges?
5. Which simulation scenarios did you use?

1. Do you have any thoughts on integrating VR simulation as part of the formal learning objectives in your first-year residency program?
2. Advantages/disadvantages

1. Can you tell us how you experienced the learning effect of using VR during your first-year residency program?
2. How did you choose which simulation scenarios to try?
3. What influenced your choice to repeat a particular simulation scenario or not?

1. Would you recommend the use of VR simulation to the next group of first-year residents?

# Appendix 2

Questionnaire

**Gender**

- Female
- Male
- Other

**Age**

- <30 years
- >30 years

**First-year residency program period**

- I will complete the hospital program in August 2023
- I will complete the hospital program in February 2024

**Have you previously used VR in an educational or work-related context?**

- Yes
- No
- If Yes, please specify: [free text]

**Have you used VR in other contexts (e.g., gaming)?**

- Yes
- No
- If Yes, please specify: [free text]

**How many hours have you spent using VR simulation during your first-year residency program (approximately)?**

- 0 hours
- 1-4 hours
- 5-10 hours
- 15-20 hours
- 20 hours or more
